# Patients’ and Nurses’ Perceptions of Diabetes Self-Management in Oman: A Qualitative Study

**DOI:** 10.3390/ijerph19116929

**Published:** 2022-06-06

**Authors:** Abdullah Salim Al Mahrouqi, Robert Kevin Mallinson, Kyeung Mi Oh, Ali A. Weinstein

**Affiliations:** 1 Oman Government Ministry of Health, Oman College of Health Sciences, Ibri 516, Oman; 2 School of Nursing, George Mason University, 4400 University Dr, Fairfax, VA 22030, USA; rmallins@gmu.edu (R.K.M.); koh5@gmu.edu (K.M.O.); 3 Department of Global and Community Health, George Mason University, 4400 University Dr, Fairfax, VA 22030, USA; aweinst2@gmu.edu

**Keywords:** patient-centered care, self-management, diabetes, nurses, patients

## Abstract

Patient-centered care enhances diabetes self-management; however, the primary care nurse’s role in promoting diabetes self-management within a patient-centered care model is unexplored. This study investigated the perceptions of Omani patients with type-2 diabetes and their clinic nurses on the nurses’ role in promoting diabetes self-management within a patient-centered care approach. The thematic analysis of the data from individual interviews with patients (n = 24) revealed two themes: patients experienced “missteps on an unclear path” and “nurses doing their best.” Patients struggled to identify treatment goals and faltered in their attempts to adopt diabetes self-management behaviors. The nurses’ role was perceived as task-oriented. Nurse narratives (n = 21) revealed that very few nurses were aware of the patient-centered care philosophy. A theme emerged of nurses “needing a new perspective” to transition their care delivery to align with the patient-centered care model. Nurses expected patients to comply with their instructions and missed opportunities for assessment, engagement, and collaborative problem-solving during patient encounters. The shift from a physician-based medical model to a patient-centered primary care delivery system may necessitate that nurses engage more effectively with patients, collaborate on an individual treatment plan, and motivate them to adopt self-management behaviors.

**What Is Already Known**

- Patient-centered care enhances diabetes self-management.
- Patient-centered care studies have focused on how physician–patient relationships affect health outcomes.
- Scant literature describes the primary care nurse’s role in promoting diabetes self-management within a patient-centered care model.

**What This Paper Contributes**

- Patient perceptions that nurses have a limited role in diabetes management may alert managers to ineffective clinical encounters that lack patient engagement, collaboration, and problem solving.
- Primary care nurses need to assess patient beliefs and self-management behaviors to develop individual plans for achieving optimal health outcomes.
- Task-oriented nurses with little understanding of patient-centered principles may fail to educate and motivate patients to adopt effective diabetes self-management behaviors.

## 1. Introduction and Background

Approximately 463 million adults in the world live with diabetes mellitus (DM), and the number is estimated to reach 629 million by 2045 [1]. Diabetes is a rapidly increasing health problem in developing countries such as Oman [2]; in particular, type 2 diabetes (T2DM) is an emerging public health issue for the country [3]. The reported prevalence of diabetes among Omanis is 15.7% and, along with other chronic diseases, causes the death of 187.75 people per 100 thousand in Oman each year [4]. Therefore, urgent attention is needed to address this significant burden on Oman’s healthcare system. Research suggests that the changing lifestyle of the Omani population has led to increased obesity that contributes to the rising cases of T2DM [5]. Omanis are engaging in less physical activity while consuming more sugar and saturated fats; their diet lacks sufficient fiber [6]. By tradition, Omani people consume unhealthy foods—sweets, sugary beverages, and high-carbohydrate foods—at cultural and social events. Many Omani patients with T2DM inconsistently perform diabetes self-management behaviors [7].

Ineffective diabetes management contributes to preventable morbidity and mortality, decreased quality of life, and increased healthcare costs [8]. Patients living with T2DM must have the ability to self-manage their condition early, potentially improving their health outcomes [6]. The majority of patients with T2DM are more likely to adhere to taking medication than engaging in other diabetes self-management (DSM) behaviors [9]; however, medication compliance alone may be insufficient to achieve optimal healthy outcomes [10].

Researchers have suggested that promoting DSM behaviors may be more successful if healthcare systems adopt a patient-centered care (PCC) model of care delivery [11]; the PCC approach emphasizes collaboration between healthcare providers and their patients [12,13]. Although PCC may be considered a gold standard for quality health care, most PCC studies have focused on how physician–patient relationships affect health outcomes [14]. In primary care diabetes clinics, nurses contribute to the PCC model of care delivery and promote DSM with patients [15] through effective nurse–patient relationships [16]. The effective nurse–patient relationship has been significantly correlated with patients’ positive health outcomes [17].

The Omani Ministry of Health (MOH) recommended integrating PCC principles into its healthcare system to address chronic conditions [18]. The report underscored that the transition to a PCC model in Oman will require engaging nurses as members of the primary care team who can deliver effective health promotion and patient education [18]. The literature is silent on the role of the primary care nurse in promoting DSM in a PCC model. Scant literature describes how nurse–patient relationships affect health outcomes in primary care settings [19,20]. Research that focuses on outcomes that are sensitive to the nurse–patient relationship is needed [16]. This paper reports on the qualitative findings from a mixed-methods study that explored PCC and DSM of adult patients with type 2 diabetes in Oman.

### Study Purpose and Questions

The purpose of this study was to explore perceptions of PCC and DSM by both patients and their nurses in primary healthcare (PHC) clinics in the Sultanate of Oman. Three research questions were addressed: (1) *What are the patient perspectives of the PHC nurses’ role in facilitating self-management of their type 2 diabetes?* (2) *What are the nurses’ perceptions of their role in facilitating DSM for their patients?* and (3) *What are the nurses’ perceptions of PCC as it applies to their role in the diabetes clinic?*

## 2. Methods

### 2.1. Research Design

This qualitative component of a mixed-methods study examined the perceptions of patients and nurses. Employing a descriptive, qualitative design, we conducted unstructured, individual interviews with patients with T2DM and their PHC clinic nurses. The use of an exploratory design was appropriate to gain insights into the perceptions of patients and their primary care nurses. This report aligns with the guidelines for the COnsolidated criteria for REporting Qualitative research (COREQ) checklist.

### 2.2. Sample and Setting

The sample for this qualitative component consisted of two populations: patients and nurses. Patient participants were a subset of survey respondents (N = 237) who voluntarily provided their names on a questionnaire to indicate their willingness to engage in a face-to-face interview. Eligible patients were Omani citizens, 18 years or older, and diagnosed with T2DM for at least one year; they were registered and attended diabetes clinic appointments. The clinic nurses assured that no patient with a history of cognitive dysfunction, mental health condition, or sensory impairment that could interfere with effective discourse would be offered an interview. The interviewer assessed the global cognition of each participant with a screening question: “Tell me, how did you come to the clinic today?” No patient was excluded from participation. Participants were recruited from diabetes clinics in each of five governorates in the country (i.e., Muscat, North Al-Batinah, Al-Dhahira, North Ash Sharqiyah, and Dhofar). Recruitment continued until data saturation was achieved, namely, the point at which subsequent interviews provided no new insights relevant to the research questions.

Nurses were eligible to participate in an interview if they worked directly with patients in the clinic. Study flyers were posted in the nursing office and staff cafeteria at each clinic with permission. Nurses interested in being interviewed contacted the researcher to schedule a face-to-face interview.

Before conducting this study, ethical approval was received from the Centers of Studies and Research in the Omani Ministry of Health [MOH/CSR/20/23583] and the George Mason University Institutional Review Board [#1618529-1]. The study procedures were explained to the nursing staff and nurse-in-charge at each clinic. All participants signed an informed consent document before their interview commenced.

### 2.3. Data Collection

Each participant, whether a patient or nurse, was individually interviewed in a private room in the diabetes clinic to provide privacy and minimize interruptions. The first author, an Omani male nurse and PhD student, conducted all interviews. After obtaining informed consent, the interviews were conducted in Arabic and audio-recorded; the audio-recording was used to provide an accurate data record and allowed for subsequent transcription. The face-to-face, iterative interviews averaged 20 min each, and none were repeated.

Patient interviews began with the main question: *How do nurses help you manage your diabetes so you can stay healthy?* Standard qualitative probes were used to elicit elaboration, clarification, and sequencing in the narratives. The nurses’ interviews began with the PCC question: *The MOH has been promoting the implementation of PCC in health services. What does PCC mean to you?* Probes were used to encourage the nurses to expound upon their responses before being asked their second question: *What is your role in helping patients manage their diabetes*? The use of standard qualitative probes helps the researcher to focus the interview and obtain comprehensive data. After each interview, the interviewer recorded field notes and reflective notes to enhance data analysis.

Every effort was made to interview patients while they waited to see the physician. The nurse interviews were conducted outside busy clinic hours to avoid interrupting workflow. When each interview was completed, participants were offered an opportunity to pose questions or concerns to the interviewer. Each participant was compensated for their time with either a phone card or a small souvenir (worth approximately USD 8).

### 2.4. Data Analysis

The audio recordings were translated from Arabic into English during the process of transcription by the first author. The English translations were reviewed and independently verified by a bilingual, native Arabic speaker to ensure conceptual equivalency. The transcribed files were imported into a software package for qualitative data management and analysis (Dedoose, v.8). Strauss and Corbin’s procedures for qualitative data analysis guided the coding process [21]. Relevant words and phrases were highlighted in open coding, and chunks of data were collected into categories such as “medication adherence”, “poor nursing engagement”, or “assessment practices”. The categories were expanded or collapsed during axial coding, and the relationships between categories were identified. Clinical and demographic details from the patients’ survey were reviewed to interpret individual narratives. Finally, the overarching themes relevant to the research questions about PCC and DSM were identified.

## 3. Results

### 3.1. Sample Demographics

The demographic characteristics for the patients (n = 24) are listed in Table 1; the patients were relatively young (*M* = 41.2 years ± 9.2) middle-aged adults, nearly evenly divided by gender. A large majority reported having 12 years or less of education. More than two-thirds of the sample were married, and the majority of them had a monthly household income of 1000 Omani Rial (OMR) (~USD 2600) or less. Nearly two-fifths of the sample were prescribed oral medication and insulin therapy for their diabetes. Most of the nurse participants (n = 21) were female and, on average, 37 years of age (± 5.4). A large majority of the nurses held a general diploma degree, and four nurses had earned a bachelor’s degree or higher. The nurses were experienced (*M* = 16 years ± 5.0, range 7–24 years). The patient and nurse sample sizes were relatively equivalent across the five PHC diabetes clinics, representing each of the five Omani governates. More Demographic info can be found in Table S1.

### 3.2. Findings

Theme 1: Missteps on an Unclear Path (Patients)

When patients were asked about the nurses’ role in helping them manage their diabetes, they commonly spoke of receiving instructions from nurses about their diet, exercise, and medication compliance. However, patients were uncertain about the rationale for adopting these behaviors and were unable to identify specific goals—steps on the path—to achieve better health outcomes. Consequently, patients made errors or harbored incorrect beliefs about their disease. The theme that emerged from patient interviews was *missteps on an unclear path*.

Some patients noted that managing diabetes was dependent, at least partly, on oneself. One man (P4) acknowledged that he has a role in his care:

*The person should be responsible for his life and should not be careless about his life.*

However, his care behaviors may have been based upon misunderstanding effective interventions for controlling his blood sugar levels; he continued:

*I started to read about diabetes either through… alternative medicine, and I have some experience in this area. I started taking warm *[tap] *water, and my blood sugar readings improved. Today, my fasting blood sugar was 126 *[mg/dL]*, which is rare. I also use a mixture of sea salt, lemon, water, and a small amount of natural apple vinegar.*

Similar to other participants, this man gauged the effectiveness of his actions (e.g., “natural” remedies) on a single fasting blood sugar result and not a measure of long-term glycemic control.

All of the participants reported engaging in some degree of physical activity, dietary control, or medication compliance. Only two participants mentioned that they self-monitored their blood glucose; a single patient mentioned regular foot care. Within the first few minutes of their interviews, numerous participants admitted to inconsistent compliance with providers’ recommendations. One patient (P20) relayed her struggle:

*I became not concerned about my food; I became bored and tiresome from following the same regimen. It was a period, then I resumed the same routine. I stopped visiting the diabetes clinic for almost one year and doing lab investigations*.

[blood glucose tests]

The patients could not provide examples of how nurses helped them to adopt strategies that might effectively keep them on a path towards better health. For example, some patients struggled to balance their dietary restrictions when attending social gatherings and traditional celebrations important in the Omani culture. Without preplanning, one participant (P24) acknowledged that her dietary misstep had consequences:

*Sometimes I mess up, especially in the gathering with others; I eat from this and that. As you know, my blood sugar control becomes messy, and I have a stomach upset.*

One woman (P10) believed she was getting at least the recommended 30 min of the recommended physical activity in her home; however, the intensity of her intermittent activities was difficult to determine. She offered:

*I climb the stairs back and forth. I have work in the kitchen, clean the house...*

Most of the patients were unaware of any specific goals, or a treatment plan, to provide them with a clear path towards better outcomes; they lacked strategies to avoid inevitable missteps.

Theme 2: Nurses Doing Their Best

**Patient Perceptions**. The patients were asked about their perceptions of the role of the diabetes nurse in supporting their efforts to self-manage their disease. Most of the patients were unable to describe nursing actions that included anything more than the routine clinical tasks; rather, there were many descriptions of how nurses were *doing their best* to move patients through the clinic process. Several participants clearly described the lack of a nurse–patient relationship. One (P14) patient whose current AIC was >13% provided a perception that was repeated across most interviews:

*I do not have a chance to have a conversation with nurses; I only see doctors.*

Most frequently, participants described nurses as pleasant and welcoming, but task-oriented; for example, one patient (P17) stated:

*I did not interact with nurses too much…when I come here, they do the [blood tests] and the rest of things with doctors. However, the interaction of nurses is kind.*

After attending the diabetes clinic for 20 years, a patient (with uncontrolled diabetes) echoed:

*Indeed, nurses do nothing; only they check blood sugar and measure blood pressure. The doctor is the basis for this clinic, whereas the nurse does not have a role only taking [blood tests], and they do not provide any advice. The doctor has a significant role.*

(P14)

Patients, nonetheless, expressed general satisfaction with the pleasantness of nurses. It seemed that they had low expectations for the role of the nurses. One man (P14) offered “They do their best.”

*I found that nurses give good health care, and we are not complaining. This is their work.*

Indeed, several participants described the role of the nurse in the diabetes clinic as quite limited or irrelevant. A man (P18) with 10 years of living with diabetes offered, disappointedly:

*I do not feel they have a role in diabetes care. Maybe the nature of their work does not require them to guide you. Their role is to perform what doctors delegate them to do.*

He continued, offering an example what a nurse *should* do to help a patient manage their diabetes, suggesting an interactive discourse with patients to explore their challenges:

[If] *they find high blood sugar? They* [should] *ask the patient what he has eaten lately?*

Whereas some patients described their nurses as being respectful, cooperative, and humble, there was little said about nurses being helpful. The participants—many of whom have had diabetes for years—struggled to describe how nurses support them in managing diabetes. Their responses were replete with comments about nurses preparing them to see their physician.

Most of the patients reported basic, unidirectional nursing actions—advising, instructing, informing—telling them how to manage their diabetes. Often, the information was described as generic and lacking evidence of patient engagement or empowerment. The nurses’ advice informed patients in a directive manner about what they *should* do to control their diabetes. Patients provided no examples of nurses collaborating with them on an individualized plan to address the challenges they face in managing their diabetes. For example, one patient (P9) noted:

*Nurses provide us with some advice about food, be away from sugary food, do physical activity whenever possible, and follow diet control.*

Some patients recalled nurses warning that noncompliance with their advice may lead to morbidities and stressing how compliant patients could circumvent adverse outcomes:

*Nurses advise you…inform you regarding the complications of diabetes and the preventive measures to avoid these complications.*

(P21)

However, there were also patients who perceived such forewarnings to be poorly timed, fear-based messages that were negative and demotivating at times when they needed positive support. It is notable that there was little variation in the patient perceptions within—and across—the five study sites. The patients did not seem to expect much support for self-management from nurses. While perceived to have a limited role, patients thought nurses were *doing their best*.

**Nurse Perceptions.** In alignment with the patients’ narratives, most nurses reported *doing their best* to care for patients. They described completing clinical tasks (e.g., vital signs), advising patients, and encouraging compliance. One nurse (Nur11) explained:

*My role in the diabetes clinic is to receive patients, check their blood sugar, guide them to their responsible doctor. I also provide any help, instruct them on the importance of taking care of their health and maintaining* [a healthy] *blood sugar level.*

Indeed, fewer than one-third of the nurses described actions beyond the routine clinical tasks; most nurses only provided a single example of an intervention to promote patient self-management. One nurse (Nur2) stood out from the others by describing his individualized assessment and problem-solving approach:

*We question the patient about his eating habits and lifestyle to know how he spends his life. We are trying to know what the problem is. If we find the problem, the problem is that eating, lifestyle, life circumstances, or psychological factors impact him. Based on the problem, we will try to find the right way to help the patient.*

Although lacking assessment and planning, a few nurses reported sitting with patients, listening to problems, and offering solutions.

One experienced nurse had attended workshops on diabetes management; she was one of the few who seemed to grasp the complexities of behavioral change. She (Nur5) described a “harm reduction” approach to helping a patient with dietary challenges:

*We sit and listen to the patient “Why is his blood sugar like this?” We see his nutritional history, what he likes to eat. “I like to eat dates.” It is okay, we do not like to deprive him of eating dates, but we ration the amount he can eat per day. The first point is to ration his food. The second point is not only telling him and leaving, but to follow-up with him.*

This was the only comment suggesting that nurses would need to evaluate the patient’s adherence to treatment.

Many of the nurses described involving the patients’ family members in diabetes care. If a patient struggled, one nurse (Nur10) would engage the family at the next clinic appointment:

*Involving patients’ families may support patients at home because, after a time, the patient may not follow the required treatment plan by himself. If the family does not eat a healthy diet, the patient eats what they want. We encourage families to follow a healthy lifestyle to encourage patients to do the same.*

In summary, the inability of most nurses in this study to clearly explicate their role in supporting DSM aligned well with the perceptions provided by the patients. Only a few of the nurses were able to describe an appreciation for their patients’ challenges or motivations for behavior change. The narratives provided little evidence that nurses effectively promoted diabetes self-management among their patients.

Theme 3: Needing a New Perspective

**Nurse Perceptions of PCC.** The second research question for the nurses was to explore their understanding of the new model of care being integrated into the Omani healthcare system: *patient-centered* care. As integral members of the healthcare team, nurses could significantly contribute to the delivery of PCC to patients in these clinics. However, none of the nurses in this study expressed an awareness of the government’s initiative to integrate the PCC philosophy into care delivery. They were *needing a new perspective* on how to transition from a physician-driven model in which nurses play a supportive, but task-oriented role, to a patient-centered approach that will necessitate a significant improvement in how nurses interact with their patients.

The narratives revealed that more than half of the nurse participants were altogether unaware of the PCC concept. A common response was: “I have not heard about it.” The overwhelming majority of the nurse participants were unable to describe even the most basic elements of the PCC approach. For example, when asked about PCC, one nurse (Nur10) responded:

*Honestly, I do not know about it, unless there is another name. I have no background about it unless someone has explained it to me in another way.*

The nurse narratives had references to patients as “noncompliant”, “defaulters”, and “[patient] careless in his treatment.” Some comments revealed that nurses misconstrued PCC as the patient being responsible for their outcomes; therefore, nurses might blame the patient, rather than reflecting on their role in helping them self-manage their diabetes. One nurse (Nur2) noted that PCC would involve a focus on the patient and, therefore, incorrectly placed the responsibility for outcomes solely upon the patient:

*The healthcare that depends on the patient by himself, in the way he interacts with the disease, as he is the cornerstone of the treatment plan.*

Only 4 of the 21 nurses in the interviews were able to identify even a basic tenet of the PCC approach. Although not sounding confident in her understanding of PCC, one nurse (Nur7) suggested the individualized approach to patient care might be a possible element of the PCC concept:

*Maybe you mean that each patient is an individual? Meaning that each patient has his treatment plan that differs from another patient?*

Another nurse (Nur5) seemed to be developing a *new perspective*, highlighting the healthcare providers’ responsibility to conduct a holistic assessment before designing interventions for that person:

*It means the patient is the* [focus of] *healthcare and we care for the patient as a whole. We work, develop ourselves, and improve healthcare services. Why? Only for patients. When we study the patient from all aspects, we will know the deficits or shortcomings to improve them, potentially affecting the patient’s quality of care that is provided.*

Another nurse (Nur1) described PCC in terms of nurse–patient engagement, discussing treatment options, and providing the patient with the requisite information to make an informed choice.

*Engage patients with suggestions and the methods of treatment that they need to follow. Give [them] the priorities to choose their treatment and provide an in-depth explanation about the chosen treatment, such as medication side effects and the best for them.*

The nurse participant with the most years of experience described how she sought patients’ “adherence”, rather than foisting unwanted demands on the patient and enforcing their compliance. She was aware that a punitive approach could undermine the nurse-patient relationship and threaten the patient’s return to the clinic or cooperation with a treatment plan. She demonstrated the importance of taking a *new perspective* on nurses’ (Nur4) interaction with patients:

*We do not force and impose our opinions on the patients’ treatment plan. For example, we do not give patients instructions or orders (“Do this and that, take this” and “You must take this”). Even in diet control, we give patients choices from their daily meal plan, but we reorganize the diet for them (“take this, instead of that”). We do not want the patient to reach the stage of refusal, and we lost the patient.*

Generally, the few nurses who had the most experience appreciated some of the basic tenets of PCC; they spoke of respect for the patients’ autonomy and individuality. Despite being unaware of the Ministry of Health’s move towards PCC, these few nurses were beginning to evidence a *new perspective* on patient encounters. They saw their role as meeting patients’ needs, not enforcing patient compliance. The four nurses with a bachelor’s preparation or post-graduate training demonstrated no more awareness of patient-centered care than the diploma graduates.

## 4. Discussion

Most patients in this study recognized their personal responsibility for adopting self-management behaviors and noted personal and social barriers to behavior change. Few offered self-monitoring of blood glucose or regular foot care as examples of personal DSM behaviors, similar to reports from patients in Iran [22]. Although clinic services are provided in Oman at no cost to patients, patients are responsible for purchasing supplies for home blood glucose monitoring; limited finances may be a barrier to self-management [23,24]. Some patients felt that nurses had little—or no—role in helping them self-manage their diabetes. Nurses were generally perceived as warm and socially welcoming, but mostly performing a limited array of basic tasks. As other researchers have reported, physicians were perceived as the center of diabetes management, with nurses having a dependent role, responsible for carrying out physician orders [25].

Our findings align with a previous study in Oman [26] that described nurses as providing didactic advice, instruction, and guidance to their patients. The narratives in our study were nearly devoid of references to patient engagement, collaboration, partnership, or problem solving, all of which may be characteristic of a patient-centered approach that encourages effective behavior change for optimal self-management. Our patients perceived nurses as “*doing their best*” even when their actions were task-oriented and lacked professional engagement; this is in stark contrast to how some authors characterized nurses as “going the distance” in their efforts to establish effective nurse–patient relationships [16]. The majority (80%) of the clinic nurses in our sample had completed only a diploma education in nursing; it may be that their educational programs focused on completing tasks and procedures and not independent practice.

In our study, the nurses’ perceptions of their role in helping their patients self-manage their diabetes were strikingly similar to the perceptions of their patients. Their interviews were replete with performing clinical tasks and advising patients on dietary control. Otter and colleagues described how nurses with a limited understanding of the self-management concept attempted to persuade patients to follow their advice [27]. Traditionally, the medical model of care was founded upon patient compliance [28] rather than patient adherence to a collaborative plan of action [29]. Our nurses did not discuss approaches central to the nurses’ role in supporting DSM behaviors, such as assessing patient motivations [26], forming a nurse–patient relationship with empathetic and purposeful communications, or partnering with patients to meet specific goals [30].

We found very little understanding of the PCC concept, and how to implement it in practice, among the nurses in our study. This may suggest that Omani nurses have not yet received adequate orientation and training in this new model for patient care. The PCC approach is central to effective patient self-management because it requires provider–patient encounters characterized by holistic assessment and goals of therapy that are tailored to the individual [30,31]. Our data suggest that nurses are not functioning in a role that effectively supports patient self-management.

The trustworthiness of these findings was strengthened by the researcher’s prolonged engagement on-site at each diabetes clinic; the researcher witnessed nurse–patient encounters that were consistent with the narrative findings. The dependability of the findings was strengthened by having a nurse expert in qualitative research (co-author RKM) oversee the analysis process and audit trail to ensure that decisions were authentic, confirmable, and justifiable. Additionally, an independent native Arabic speaker verified the translation from Arabic audio to the English transcription before analysis commenced. The trustworthiness of the findings was enhanced with the collection of data from both patients and their nurses; the two sets of data allowed for triangulation from the various perspectives. The transferability of the findings in-country may be strengthened by the recruitment of participants from various regions across Oman. These findings, however, have relevance for healthcare systems in other countries that are in transition from a traditional medical model to a patient-centered delivery care model, and systems with an abundance of nurses with a minimal level of education.

### 4.1. Limitations

It is possible that both the patient and nurse participants were unfamiliar with expressing their opinions in face-to-face research interviews, leading to superficial responses. Furthermore, participants may have provided socially desirable answers to the researcher’s questions. Researchers may consider conducting follow-up interviews to allow participants familiarity with the process, time for reflection, and rapport with the researcher.

### 4.2. Implications of the Findings

The findings of this study suggest that the awareness of PCC principles among the nurses in the diabetes clinics may be limited, impeding their ability to support patient self-management behaviors. If the vision for the future of Oman’s healthcare system is to be realized, all healthcare team members must appreciate the PCC philosophy and be able to demonstrate it within their scope of practice. Administrators responsible for implementing the PCC model are encouraged to design in-service trainings that provide nurses with the requisite knowledge and skills to engage in dynamic patient encounters characterized by relationship building, effective communication, and collaborative goal setting with patients. To prepare for the future, educators may use our findings to ensure that the curricula in nursing programs embrace the PCC approach and prepare graduates to function at the level of their professional education.

It is recommended that implementation of the PCC model in primary care involves patient orientation and empowerment. As stakeholders, patients can provide valuable input into effective strategies to improve care delivery. Researchers may consider conducting ongoing evaluations with both patients and healthcare workers as the nationwide implementation of the PCC model unfolds.

In conclusion, neither patients nor nurses demonstrated an understanding of the patient-centered approach or theory-based strategies for patients’ self-management of diabetes. Transitioning the Omani healthcare system to a patient-centered model of care that engages patients to improve their health outcomes will require that nurses be full participants in the healthcare team. Nurses may require focusing on orientation and continuing education for their role in promoting patient self-management of chronic diseases in a patient-centered care model. Ultimately, the goal is to optimize the patients’ quality of life, reduce diabetes morbidity and mortality, and lower healthcare expenditures.

### 4.3. Relevance to Clinical Practice

The delivery of patient-centered care requires primary care nurses to go beyond the performance of basic tasks to effectively engage with patients. Researchers in countries whose healthcare system is based on a medical model may need to evaluate how their nurses are prepared for more than rote duties; such an assessment may be particularly necessary when the majority of primary nurses have completed only a diploma program. Our findings suggest that some nurses may not conduct a thorough assessment of the patient with T2DM to appreciate how their individual beliefs and unique challenges may affect the adoption of self-management behaviors. Nurse leaders in primary care settings are encouraged to assess how nurses are implementing a patient-centered care model and provide orientation and supplemental training as needed. Data may be collected through regular patient surveys and interviews to evaluate the impact of nurse encounters on patients’ self-management of their chronic conditions. Nurses that lack an appreciation for evidence-based behavior change approaches may fail to motivate patients with chronic conditions to initiate—and maintain—self-management behaviors. The patient-centered approach may be crucial to assisting patients in achieving optimal health outcomes.

## Figures and Tables

**Table 1:** Demographic characteristics of the interview participants.

Demographics	Patients (n = 24)	Nurses (n = 21)
	M ± (SD)	
Age (years)	41.2 ± 9.2	37.1 (5.4)
	range: 25–60	range: 28–49
Years of experience	-	16 ± 5.0
	n (%)	n (%)
Male	13 (54.20)	19 (90.5)
Female	11 (45.80)	2 (9.5)
Did not complete 12th grade	4 (16.7)	-
Completed 12th grade	9 (37.5)	-
Diploma	4 (16.7)	17 (81.0)
Bachelor	2 (8.3)	3 (14.3)
Post-graduate	5 (20.8)	1 (4.7)
Marital Status	4 (16.7) 17 (70.80) 2 (8.3) 1 (4.2)	-
Unmarried		
Married		
Widowed		
Divorced		
Medication Type		-
No medication	2 (8.3)	
Pills only	8 (33.3)	
Insulin only	5 (20.8)	
Pills and insulin	9 (37.5)	
Monthly Income (OMR)		-
Below 500	7 (29.2)	
501–1000	10 (41.7)	
1001–1500	5 (20.8)	
More than 2000	2 (8.3)	
PHC site of care		
Al-Dhahira	5 (20.8)	4 (19.0)
North Al-Batinah	6 (25.0)	4 (19.0)
Dhofar	4 (16.7)	4 (19.0)
South Ash Sharqiyah	5 (20.8)	4 (19.0)
Muscat	4 (16.7)	5 (24.0)

## Data Availability

The data that support the findings of this study are available from the corresponding author upon reasonable request.

## Supplementary Materials

The following supporting information can be downloaded at: https://www.mdpi.com/article/10.3390/ijerph19116929/s1, Table S1: Demographic.
