# Supplementary material for: Patients’ and Nurses’ Perceptions of Diabetes Self-Management in Oman: A Qualitative Study
Source: Int J Environ Res Public Health. 2022 Jun 6;19(11):6929. doi: 10.3390/ijerph19116929 (PMC9180150; doi:10.3390/ijerph19116929)
Supplement: Supplementary file 1 [file ijerph-19-06929-s001.zip › ijerph-1730944-Table S1.pdf]

Supplementary Table for Demographic

Patient Participants	Age	Gender	Diabetes Duration	HbA1c	Nurse Participants	Age	Gender	Years of Experiences	Education Level
P1	37	Female	6	7.5	Nur1	49	Male	24	Diploma
P2	60	Female	21	5.5	Nur2	39	Female	20	Diploma
P3	46	Male	6	6.7	Nur3	35	Male	15	Bachelor
P4	48	Male	8	8.9	Nur4	48	Female	24	Diploma
P5	25	Male	2	10.2	Nur5	41	Female	21	Diploma
P6	50	Male	8	11.0	Nur6	39	Female	20	Diploma
P7	59	Male	3	8.0	Nur7	30	Female	9	Diploma
P8	35	Male	16	11.6	Nur8	34	Female	13	Diploma
P9	35	Female	3	5.5	Nur9	39	Female	18	Diploma
P10	42	Female	12	7.7	Nur10	28	Female	7	Diploma
P11	50	Female	23	7.7	Nur11	29	Female	8	Diploma
P12	32	Female	12	9.4	Nur12	29	Female	8	Diploma
P13	33	Male	3	6.1	Nur13	38	Female	16	Diploma
P14	33	Female	15	13.4	Nur14	38	Female	19	Diploma
P15	38	Male	1	6.3	Nur15	38	Female	17	Diploma
P16	45	Female	4	13.2	Nur16	38	Female	17	Diploma
P17	52	Male	21	9.0	Nur17	36	Female	15	Diploma
P18	43	Male	10	7.5	Nur18	39	Female	16	Bachelor
P19	48	Female	19	8.7	Nur19	34	Female	13	Bachelor
P20	34	Female	8	7.8	Nur20	40	Female	20	Diploma
P21	40	Male	11	8.2	Nur21	36	Female	16	Post-graduate
P22	28	Male	7	7.8	-	-	-	-	-
P23	35	Male	8	8.7	-	-	-	-	-
P24	41	Female	10	10.9	-	-	-	-	-
